# Evaluation of the Performance of Newborn Screening for Tyrosinemia Type 1 in The Netherlands: Suggestions for Improvements Using Additional Biomarkers in Addition to Succinylacetone

**DOI:** 10.3390/ijns11020035

**Published:** 2025-05-09

**Authors:** Marelle J. Bouva, Allysa M. Kuypers, Evelien A. Kemper, Rose E. Maase, Annet M. Bosch, Francjan J. van Spronsen, Annemieke C. Heijboer, M. Rebecca Heiner-Fokkema, Sandra G. Heil, Anita Boelen

**Affiliations:** 1 Centre for Health Protection, National Institute for Public Health and the Environment (RIVM), 3721 MA Bilthoven, The Netherlands; rose.maase@hotmail.co.uk (R.E.M.); sandra.heil@rivm.nl (S.G.H.); 2 Amsterdam Gastroenterology Endocrinology Metabolism, 1105 AZ Amsterdam, The Netherlands; a.m.bosch@amsterdamumc.nl (A.M.B.); a.heijboer@amsterdamumc.nl (A.C.H.); a.boelen@amsterdamumc.nl (A.B.); 3 Section of Metabolic Diseases, Beatrix Children’s Hospital, University of Groningen, University Medical Center Groningen, 9700 RB Groningen, The Netherlands; a.m.dijkstra@umcg.nl (A.M.K.); f.j.van.spronsen@umcg.nl (F.J.v.S.); 4 Department of Clinical Chemistry, IJsselland Hospital, 2906 ZC Capelle aan den IJssel, The Netherlands; ekemper@ysl.nl; 5 Department of Pediatrics, Division of Metabolic Diseases, Emma Children’s Hospital, Amsterdam UMC, University of Amsterdam, 1105 AZ Amsterdam, The Netherlands; 6 Endocrine Laboratory, Department of Laboratory Medicine, Amsterdam UMC, Vrije Universiteit Amsterdam, 1081 HV Amsterdam, The Netherlands; 7 Endocrine Laboratory, Department of Laboratory Medicine, Amsterdam UMC, University of Amsterdam, 1105 AZ Amsterdam, The Netherlands; 8 Laboratory of Metabolic Diseases, Department of Laboratory Medicine, University of Groningen, University Medical Center Groningen, 9700 RB Groningen, The Netherlands; m.r.heiner@umcg.nl

**Keywords:** dried blood spot, newborn, neonatal screening, succinylacetone, tyrosinemia type 1, The Netherlands, sensitivity, positive predictive value

## Abstract

Currently, Dutch newborns are screened for tyrosinemia type 1 (TT1) using succinylacetone (SA) as the biomarker. Although the sensitivity of the test is high, a high number of false positives is observed. Here, the aim is to evaluate the current Dutch newborn-screening protocol and to assess alternatives, specifically the use of biomarkers that are already being measured, to increase the positive predictive value (PPV). TT1 screening was performed with the Revvity NeoBase assay between 2008 and 2017, and since 2018, the Revvity NeoBase 2 assay has been used. Data from 2018 to 2021 were used for evaluation. To simulate alternative screening protocols, these data were enriched with results of referrals from other periods and a false negative (FN) from 2010. In 2018–2021, 693,821 newborns were screened, resulting in 23 referrals, of whom two were TT1 patients. For this period, to date, no FN have been reported, resulting in a provisional sensitivity of 100%, a specificity of 99.997%, and a PPV and negative predictive value of 9% and 100%, respectively. To improve the PPV, we combined SA, tyrosine (tyr), tyr × SA and tyr/phenylalanine and achieved a PPV of 72% for this dataset without introducing FN in the original dataset. This illustrates that future screening for TT1 may benefit from the addition of these biomarkers.

## 1. Introduction

The nationwide newborn-screening (NBS) programme in the Netherlands started in 1974 with screening for phenylketonuria (PKU). Since then, the number of diseases screened for in the Dutch programme has expanded to the current 27, with a notable number added following the introduction of mass spectrometry in the screening laboratories in 2007. One of the essential tasks of the NBS community is to evaluate the performance of the current screening protocol for a specific disease and, if needed, to improve the performance by decreasing both the number of false negatives (FN) and the number of false positives (FP).

**Figure 1:**
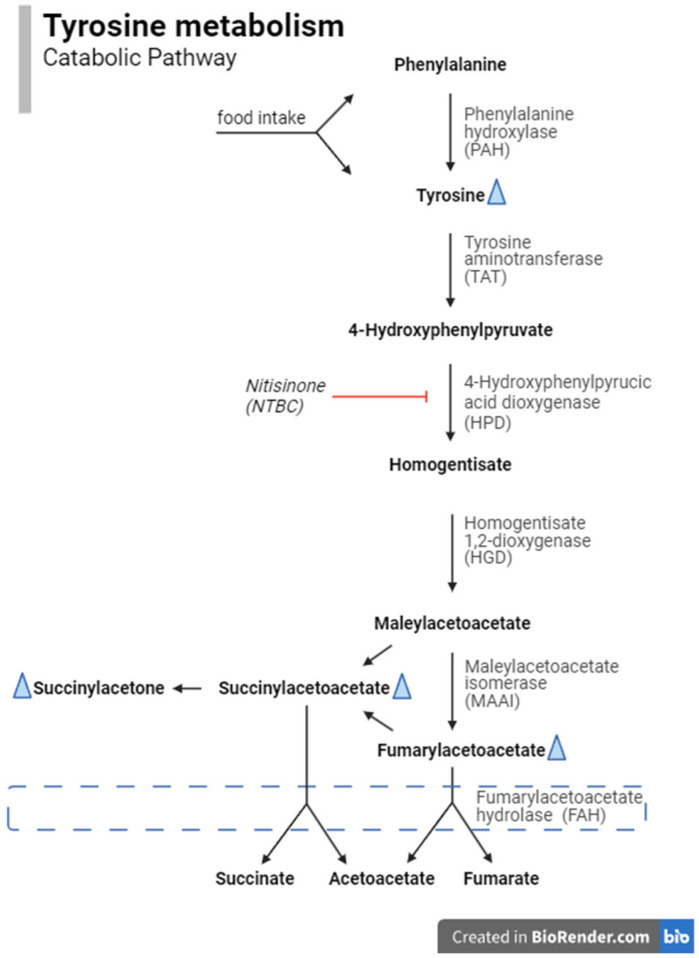
The catabolic pathway of phenylalanine and tyrosine. Biomarkers in this pathway that are analysed in the newborn-screening program are phenylalanine, tyrosine and succinylaceton. The latter two are increased in TT1 patients (indicated with a blue triangle), but TT1 patients without elevated concentrations have also been reported [5,6,7]. The blue dashed box indicates the affected enzyme in TT1, i.e., fumarylacetoacetate hydrolase.

One of the diseases screened for in the Dutch NBS programme is tyrosinemia type 1 (TT1; OMIM #276700). TT1 is a rare inherited autosomal recessive metabolic disease of tyrosine (tyr) catabolism (Figure 1) caused by DNA variants in the gene encoding fumarylacetoacetate hydrolase (FAH). Tyr is absorbed by the body through the digestion of food or produced from phenylalanine (phe) or by protein catabolism within the body. The accumulation of toxic metabolites resulting from TT1 causes severe liver pathology and renal tubulopathy if untreated [1,2]. Increased succinylacetone (SA) levels in blood and urine are considered pathognomic for TT1 but are also observed in maleylacetoacetate isomerase deficiency [3], whereas tyr may also be increased by several causes other than TT1 [4]. However, TT1 patients with normal or only slightly elevated SA or tyr values have also been reported [5,6,7]. Early detection allows timely treatment, thereby decreasing the risks for liver failure, hepatocellular carcinoma (HCC), renal tubulopathy and neuropathy and in this way considerably improving outcomes for TT1 patients [8]. TT1 patients may present with classic symptoms like liver failure during the first months of life. However, it is important to note that some milder cases may manifest with HCC only after months or years [9,10].

TT1 was incorporated into the Dutch NBS programme on 1 January 2007, with initial testing using tyr in dried blood spots (DBSs) as a biomarker with a cut-off value (COV) of 500 µmol/L blood. However, after two months, screening for TT1 was temporarily suspended due to a higher-than-expected referral rate without any true-positive cases [11,12].

On 1 October 2008, a new screening protocol was implemented using SA in DBSs as a biomarker, with a COV of 1.5 µmol/L blood. A commercial assay was applied using a flow-injection analysis tandem mass spectrometry (FIA-MS/MS) method. In 2009, the COV was decreased from 1.5 to 1.2 µmol/L blood to reduce the risk of FN results, since there was one confirmed TT1 patient with an SA of 1.5 µmol/L blood and the laboratories experienced high analytical variation. Despite this preventive measure, a report was received in 2020 concerning a TT1 patient who had been missed by screening in 2010 because of an SA concentration below the COV (1.08 µmol/L blood) [10].

In 2018, the introduction of a new LC-MS/MS system and an adapted assay from the same manufacturer required reestablishment of the COV, which eventually resulted in a COV of 0.60 µmol/L blood.

Although SA is more specific for TT1 than is tyr [4], analytical challenges were encountered during determination of SA concentrations in DBSs; these challenges were similar to those reported in diagnostic assays for SA [13]. Both assays used in the Dutch NBS employ an internal standard to calculate metabolite concentrations. However, low total ion intensities may be observed for a variety of reasons, including the necessity of cleaning the MS/MS system prior to regular preventive maintenance. Low total ion intensities can result in a low signal-to-noise ratio and a transiently increased lower limit of quantitation. Subsequently, this results in falsely elevated SA measurements; such measurements were also observed around the COV, leading to an increase in the number of FP measurements. This phenomenon has been observed especially in the context of SA measurement. To enhance the specificity of the screening, the Dutch laboratories implemented re-analysis on the same DBS to confirm each result before referral in cases in which the SA concentration is above the established threshold. Furthermore, in 2021, a more rigorous preventive maintenance programme for the MS/MS systems was initiated in the Dutch screening laboratories to mitigate the effects of low total ion intensities due to instrument contamination. These measures were effective in reducing the number of FP referrals; between 1 January 2022 and 30 September 2024, nine FPs were reported [14,15]. However, besides the positive effect on the positive predictive value (PPV), reanalysis results in an increase in turn-around-time, which can delay the referral of patients in need of treatment. It also requires the use of additional DBS material, thereby reducing the quantity of blood available for the other NBS tests.

**Figure 2:**
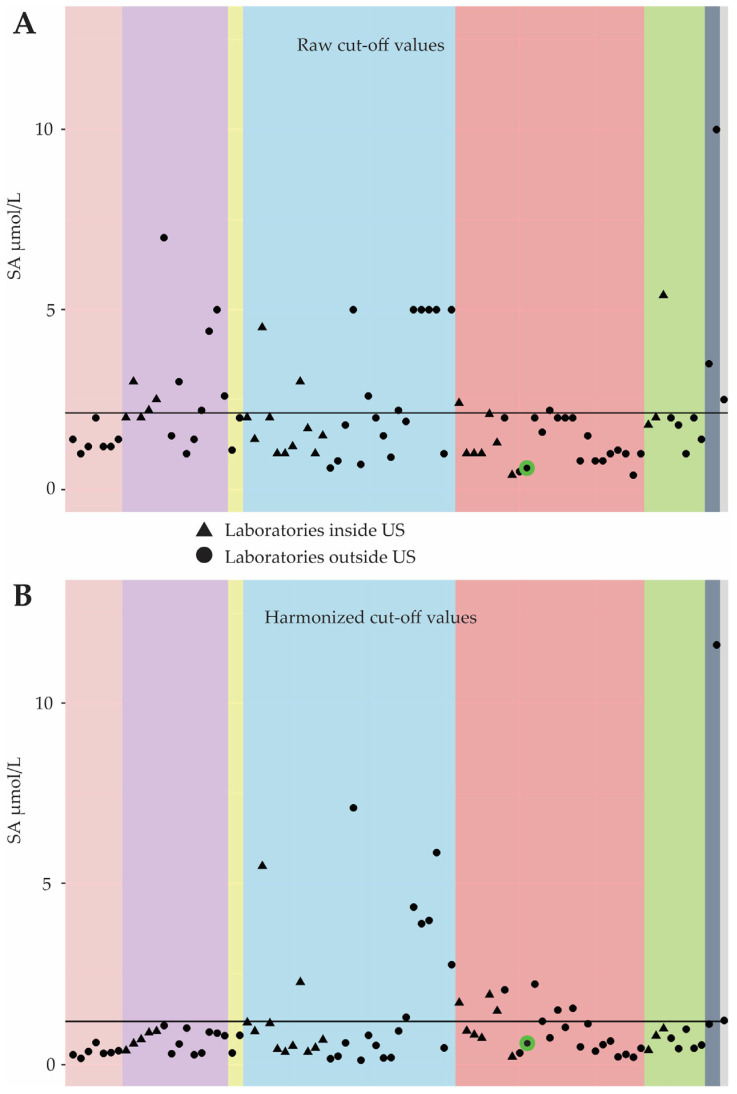
Figures comparing cut-off values for SA used in international newborn-screening programmes before (**A**) and after (**B**) harmonization. The Centers for Disease Control and Prevention (CDC) used results from the quality control (QC) samples for the Newborn Screening Quality Assurance Program. Based on the QC results from an individual laboratory and the results for the same QC samples as measured by the CDC, the CDC generated regression equations for each laboratory and metabolite, which were then used to calculate harmonized values for the proficiency samples and COV. Each colour box indicates a different MS/MS assay used to determine SA concentrations in DBSs. The red box shows the COVs of the QC programme participants evaluated using NeoBase 2. The green circle indicates the results from the Dutch reference laboratory at the RIVM. The triangles are COVs for US labs; the dots are labs outside the US. The black solid line represents the mean COV for all participants.

Kuypers et al. [16] reported a low PPV for the Dutch TT1 NBS programme compared to others using the same assay and biomarker, confirming reports from previous national monitors [14]. The Dutch COV for SA is also low, suggesting it could be increased to improve the PPV. However, Centers for Disease Control and Prevention (CDC) publications [17,18] emphasize the need for harmonization before the comparison of COVs across programmes. A comparison by the CDC shows that the harmonized Dutch SA COV is average among similar programmes (see Figure 2). Thus, modifying the COV alone may not improve the performance of the Dutch TT1 screening programme and the utilisation of additional biomarkers should be investigated. 

Considering the suboptimal performance of the TT1 screening protocol in the Netherlands, this study sought to (i) assess the efficacy of the current screening protocol, (ii) investigate potential alternative biomarkers and (iii) assess the impact of alternative screening protocols on the performance of the TT1-screening programme in the Netherlands.

## 2. Materials and Methods

### 2.1. NBS Protocol for TT1 in The Netherlands

NBS in the Netherlands is offered to all newborns (±170,000 births per year). Heel-prick blood on a DBS card was obtained between 72 and 168 h after birth for the majority of newborns (>98% [14]).

Laboratory analyses were conducted in one of the five designated regional screening laboratories in the Netherlands. For TT1 screening, SA was used as a biomarker. All laboratories followed the same nationwide screening protocol and used the same COV.

#### 2.1.1. NeoBase

Between 1 October 2008 and 31 December 2017, SA was measured using the NeoBase™ Non-derivatized MS/MS kit (Revvity (formerly PerkinElmer), Turku, Finland; further referred to as NeoBase) on the Quattro micro™ MS/MS instrument and the comparable Quattro Premier™ MS/MS instrument (Waters, Milford, MA, USA). The manufacturer’s analytical protocol prescribed two incubation steps: a 45-min incubation at 45 °C for all the biomarkers in the kit and an additional two hour to ensure complete derivatization of the extracted SA. The NeoBase procedure was conducted in accordance with the manufacturer’s analytical protocol, but in order to reduce turn-around time, the two-hour incubation for SA was omitted. The omission of the two-hour incubation step resulted in decreased SA concentrations for the analyses performed in the first two hours of the run, necessitating the application of a lower COV than might otherwise have been applied to prevent FN results. Unpublished validation experiments using DBSs of known TT1 patients showed no difference in diagnostic sensitivity with or without the two-hour incubation.

Initially, starting 1 October 2008, the COV for SA was set at 1.5 µmol/L blood. To decrease the risk of FN screening results, the COV was decreased to 1.2 µmol/L beginning on 1 July 2009. Detailed information on the different COVs and referral results can be found in Table 1.

#### 2.1.2. NeoBase 2

On 1 January 2018, the NeoBase™ 2 Non-derivatized MS/MS kit (Revvity (formerly PerkinElmer), Turku, Finland; further referred to as NeoBase 2) and the Xevo™ TQD MS/MS instrument (Waters, Milford, MA, USA) replaced the previously used method and analysis was performed according to the manufacturer’s instructions. In the event of an SA concentration above the COV, a re-analysis was conducted on one to six new extracts from the same DBS card, depending on the available DBS material, to confirm increased SA concentrations. If the increased SA concentration was confirmed, the newborn was referred. Conversely, if confirmatory testing yielded normal SA concentrations, we assumed an analytical error in the first analysis and a negative screening result was reported. If the variation between the measurements exceeded normal day-to-day variation, we suspected contamination of the DBS and requested a new heel-prick sample (repeat first-tier sample).

Following the verification of the new kit/instrument combination, it was decided that the COV should be changed to 0.90 µmol/L blood starting from 1 January 2018. Because of lower-than-expected measurement levels, as of 1 April 2019, the COV for SA was officially changed to 0.60 µmol/L blood.

#### 2.1.3. Data Registration Systems

At the screening laboratories, the results of the screening are entered into a laboratory information-management system (Neonat) designated for this purpose. Thereafter, the results are transmitted to the nationwide information system (Praeventis) from which referral is organized. In the event of a positive screening result, as well as in instances of an FN report, the screening results are registered in a national repository (Neorah). The conclusive diagnostic result for the metabolic diseases in the Dutch NBS programme is registered in a database of the metabolic centres (Dutch Diagnosis Registration Metabolic Diseases; www.ddrmd.nl) and periodically used to combine diagnostic information with the screening results in Neorah. Access to all these systems is restricted.

### 2.2. Dataset and Study Outcomes

For this study, we received approval for the use of data from the neonatal-screening research workgroup (Werkgroep Onderzoek Neonatale Hielprikscreening ‘WONHS’) and from the committee on data requests, Praeventis (Commissie Dataverzoeken Praeventis en CIMS), both entities established by the RIVM, to review requests to use residual heel-prick material and data from the Dutch NBS programme.

Pseudonymized data were obtained from Praeventis and Neorah to create a dataset (SPSS (IBM; version 29.0.1.0)) composed of all NBS results measured by NeoBase or NeoBase 2 assay and included in the Dutch NBS, including the following: diagnostic results (TT1 confirmed, yes or no), birth weight (grams), gestational age (days), gender (M/F), age at blood collection (hours) and, if applicable, information on blood transfusion. Data from screenings performed between 1 January 2022 and 30 September 2024 were added later in the study and therefore consisted of merely SA, phe and tyr concentrations, the most interesting biomarkers, and the diagnostic results from Neorah for the referred children (TT1 confirmed, yes or no). Data were used only if parents did not object (up to 2023) or if they consented (from 2023 onwards) to the use of data obtained by NBS for scientific research.

### 2.3. Performance of the Screening Protocol

Data from internal (non-public) reports produced by the reference laboratory of the NBS programme at RIVM-Centre for Health Protection were used to quantitatively evaluate the performance of the current and previous screening protocols. These annual reports review the analytical aspects of the NBS programme, including an analytical comparison of the five laboratories. For the current screening protocol, we collected data generated between January 2018 and December 2021. For the previous screening protocols, we identified three different periods based on biomarker and COV used: tyr 500 µmol/L: January–February 2007; SA 1.5 µmol/L: October 2008–June 2009; SA 1.2 µmol/L: July 2009–December 2017.

### 2.4. Alternative Screening Protocols

Data from all newborns screened between 1 January 2018 and 31 December 2021 were included in a dataset to analyse alternative screening protocols. As this resulted in the inclusion of just two TP, the dataset was enriched with TP, FP, and FN from October 2008 until December 2017 and for TT1-referred newborns from January 2022 until September 2024; these data were used to calculate a more reliable PPV. To enable integration of the data from the referred newborns across periods wherein different assays and equipment were used, SA results obtained with the NeoBase assay were converted using the method-comparison equation for SA (NeoBase2 = 0.019 + 0.572 × NeoBase, obtained via Deming linear regressions statistics using the Alternate Method Comparison module in EP Evaluator (Data Innovations LLC, Colchester, VT, USA) and based on 1093 residual NBS DBSs and quality-control samples). Based on the method comparisons for the other markers, no conversion was needed for other markers of interest. Data from TNs from 2022 until now were not available for evaluation. Data from TNs before 2018, which were analysed with the NeoBase assay, were not included in this analysis since sufficient TN data were available from the subsequent years.

In selecting biomarkers for the alternative screening protocols, we included those that were already part of the Dutch NBS programme (or that could be calculated based on those current biomarkers) and that are part of the tyrosine catabolic pathway (Figure 1). Furthermore, we used the ‘Plot by Condition’ tool in the Collaborative Laboratory Integrated Reports (CLIR) [19] as a source of information on potentially informative biomarkers. The selected biomarkers were tyr, phe, SA/tyr ratio, tyr/SA ratio and SA/phe ratio. Although they are not mentioned by CLIR, we also identified the phe/tyr ratio and tyr/phe ratio as biomarkers of interest. Furthermore, we included the tyr × SA numeric product as an equivalent to the product of immunoreactive trypsinogen and pancreatitis-associated protein, which is used in the screening for cystic fibrosis [20,21]. The COVs for the biomarkers other than SA were based on the lowest values obtained from the TT1 patients. The current SA COV of 0.60 µmol/L was maintained in the alternative protocols. The initial alternative screening protocols consisted of a single biomarker with one of the selected biomarkers. Next, alternative screening protocols with multiple biomarkers were composed; these consisted of SA as the base biomarker, with other biomarkers added one by one to create alternative screening protocols. This approach ultimately resulted in the development of 30 alternative screening protocols: those using other singleton biomarkers with various COVs (*n* = 13) and those using combinations of different biomarkers (*n* = 17). We then re-evaluated the dataset using these screening protocols. A case was classified as screen-positive if all (ratio of/product of) informative biomarkers in that particular screening protocol were above their respective COVs. Subsequently, the number of referrals, as well as the numbers of true positive (TP), FP and FN results, were calculated. Finally, the sensitivity, specificity, PPV and negative predictive value (NPV) of the current and alternative screening protocols were evaluated. The screening protocol with the highest PPV is the preferred algorithm for the Dutch programme.

## 3. Results

### 3.1. Performance of the Screening Protocol (See Table 1)

#### 3.1.1. NeoBase

Between 1 October 2008 and 31 December 2017, 1,643,579 newborns were screened for TT1. Of these, 25 newborns were referred, and nine of the referred newborns were diagnosed with TT1 (TP). Also, a report of an FN patient was obtained. This resulted in a referral rate of 0.0015% and a PPV of 36%. Based on current information, with one FN reported to date, this resulted in a sensitivity of 90%.

#### 3.1.2. NeoBase 2

Between 1 January 2018 and 31 December 2021, 693,821 newborns were screened and 23 of them were referred for TT1. Only two of the referred newborns were diagnosed with TT1 (TP), and therefore, 21 referrals were FPs. For that period, no reports of FN referrals have been received to date, resulting in a referral rate of 0.0033%, a PPV of 9% and a provisional sensitivity of 100%, based on the current information.

### 3.2. Alternative Screening Protocols

The dataset used to analyse alternative screening protocols consisted of the results for twelve TP referrals (NeoBase: *n* = 9, NeoBase 2: *n* = 3), 45 FP referrals (NeoBase: *n* = 15, NeoBase 2: *n* = 30), 1 FN (NeoBase: *n* = 1, NeoBase 2: *n* = 0) and 653,396 TNs (NeoBase: *n* = 0, NeoBase 2: *n* = 653,396). 82 samples analysed between 2018 and April 2019 had an SA ≥ 0.60 µmol/L and <0.90 µmol/L and were classified as screen-negative at that time. However, when the SA COV of ≥0.60 µmol/L was used in the analysis of alternative protocols, these samples were classified as screen-positive. In all nine TP and 15 FP referrals screened using the NeoBase assay, the converted SA concentrations were above the current SA COV (0.60 µmol/L). The converted SA concentration of the FN was also above the current SA COV (see Table 2).

**Figure 3:**
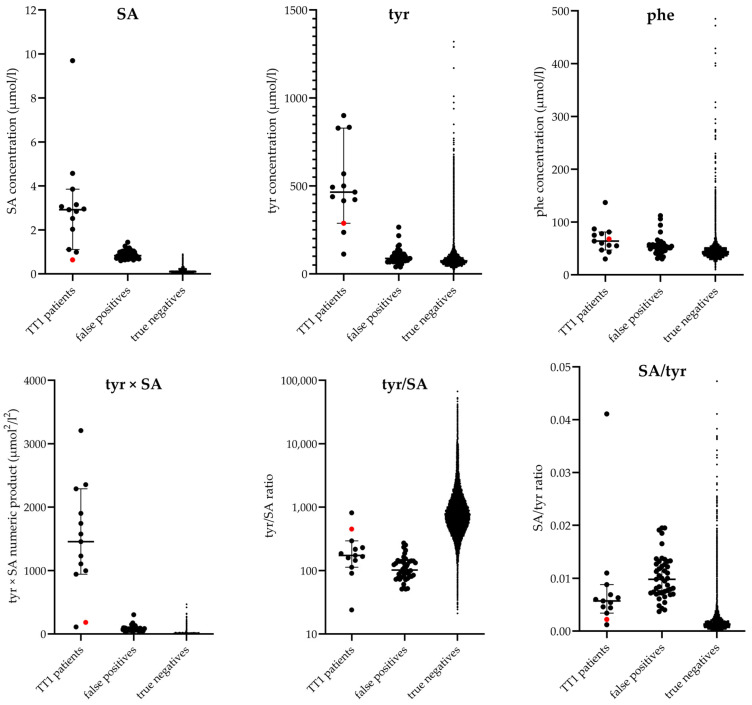

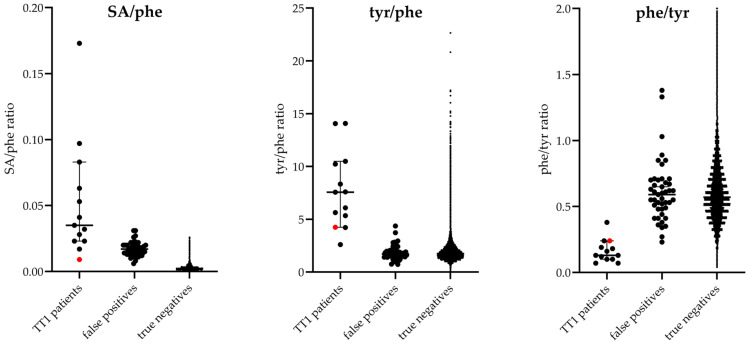
Biomarker values in enriched dataset. Overview of the considered biomarkers of interest for the screening for TT1, showing the values from the dataset used to simulate alternative protocols: 45 false-positive cases (NeoBase: *n* = 15, NeoBase 2: *n* = 30), 13 TT1 patients (12 true-positive cases (NeoBase: *n* = 9, NeoBase 2: *n* = 3) and the known false-negative case (red dot) from 2010 (NeoBase: *n* = 1, NeoBase 2: *n* = 0)) and 653,396 TN cases (NeoBase: *n* = 0, NeoBase 2: *n* = 653,396). For optimal visualization, limits are set for the y axis of the graphs showing phe and phe/tyr; therefore, some values for TNs are not shown (phe, 33 data points; phe/tyr, 1391 data points).

The following nine biomarkers were assessed in alternative protocols in various combinations: SA, tyr, phe, SA/tyr ratio, tyr/SA ratio, SA/phe ratio, phe/tyr ratio, tyr/phe ratio and tyr × SA numeric product. The values of these biomarkers for the TT1 patients, FP referrals and TNs in the dataset are visualized in Figure 3.

A total of 30 alternative screening protocols was evaluated. In 24 of the alternative screening protocols, there was no difference in the number of FN results compared to the current screening protocol, resulting in a provisional 100% sensitivity based on current information. In 15 alternative screening protocols, in addition to the sensitivities being equal, the PPVs were higher than that of the current screening protocol. For each these 15 protocols, the number of TNs, FNs, TPs and TNs; the referral rate; the provisional sensitivity; the specificity; the PPV and the NPV are shown in Table S1. The following screening protocols resulted in the three highest PPVs (Table 3): (1) a screening protocol using a combination of SA (≥0.60 µmol/L), tyr (≥100 µmol/L), tyr × SA (≥100 µmol^2^/L^2^) and tyr/phe (≥2.5) resulted in a PPV of 62%; (2) a combination of SA (≥0.60 µmol/L), tyr (≥100 µmol/L), tyr × SA (≥110 µmol^2^/L^2^) and the ratio phe/tyr (≤0.5) resulted in a PPV of 65%; and (3) a combination of SA (≥0.60 µmol/L), tyr (≥100 µmol/L), tyr × SA (≥110 µmol^2^/L^2^) and tyr/phe (≥2.5) resulted in a PPV of 72%. These three protocols detected all TPs and the known FN for an NPV of 100% and a specificity of 99.999% for this dataset. Therefore, the addition of three (ratio/product of) biomarkers increased the PPV in this dataset from 9% with the current screening protocol to 72% while maintaining high sensitivity (Table 3).

## 4. Discussion

The objective of this study was to evaluate the performance of the current screening protocol for TT1 in the Netherlands as it has been conducted since the introduction of SA as a biomarker in 2008 and to assess the performance of alternative screening protocols using biomarkers already obtained during the routine NBS for other metabolic diseases with the ultimate aim of improving the PPV of the screening for TT1. The current screening protocol in the Netherlands, which is performed using the NeoBase 2 assay, has had no known FN screening results to date (although we are aware that a false-negative result can be reported even more than 10 years after screening has taken place). Based on that information, the current screening protocol resulted in a 100% sensitivity, 99.997% specificity, 9% PPV and 100% NPV for samples collected between January 2018 and December 2021 (Table 1). Compared to the PPV obtained with screening protocols using the NeoBase assay, the PPV has declined significantly. Analysing alternative screening protocols showed that screening protocols including additional biomarkers besides SA could improve the PPV.

The present study corroborates the findings of previous research concerning the low PPV of the Dutch NBS programme for TT1 [16], which found a PPV of 22.9% between October 2008 and December 2021. During this period, eleven newborns were diagnosed with TT1 following an abnormal screening result. However, 37 referred newborns had an FP NBS result. Moreover, an FN screening result was reported in 2020 for a nine-year-old child screened in 2010, who was diagnosed with HCC due to TT1 [10]. This is currently the only reported FN screening result for TT1 in the Netherlands. This leads to an important limitation of this study: the prevalence of TT1 is low, and patients may present with symptoms, in this case HCC, at a late age, when the presentation is very different from the classic presentation that is mostly seen at a young age. If patients with such different presentations do not receive further testing to ascertain the underlying cause, TT1 may remain undiagnosed. This complicates the detection of FN and therefore impaired our study. Recently, following the publication of the FN case [10], more attention has been directed towards TT1 as a cause of HCC and other unexplained liver pathologies. This may facilitate more accurate detection of FN.

The introduction of new instruments and a new assay resulted in a significant decline in the PPV to 9% due to a combination of a lower COV compared to that of the previous method and unexpected analytical issues. Interventions, mainly in the laboratory screening protocols, appeared to have a limited impact, as the number of FP referrals decreased over time from eight in 2021, to one FP in 2022 and two FP referrals in 2023 but increased again to six FP referrals in the first nine months of 2024 [14,15]. Moreover, the mitigating measures are quite labour-intensive, with increased instrument downtime due to preventive maintenance, delayed referral of TT1 patients due to the time required to perform the repeat analysis and increased use of the limited NBS DBS sample. A screening protocol that would reduce the number of FP reports without the need to repeat analysis and/or heel-prick sampling, thereby allowing referral of TT1 patients without delay, would be preferable.

In the search for potentially useful biomarkers, this study was limited to the metabolites that are part of the tyr catabolic pathway affected by TT1 (Figure 1). In addition to SA and tyr, phe, the ratios of SA/tyr, tyr/SA, SA/phe, phe/tyr and tyr/phe, and the numeric product of tyr × SA were considered. Tyr × SA appeared to be an interesting biomarker combination that has not been studied before and that is equivalent to the potential use of the product of immunoreactive trypsinogen and pancreatitis-associated protein in the screening for cystic fibrosis [20,21]. Both Tyr and SA are not 100% specific for TT1 at itself, but, theoretically, multiplying the measured concentrations could result in a more specific biomarker combination for TT1 screening.

Initially, alternative screening protocols were simulated using data from newborns screened between January 2018 and December 2021 using the NeoBase 2 assay. However, the dataset included only two TT1 patients, and thus, there was too little power to allow us to draw conclusions. Therefore, we also requested data from newborns referred after screening between January 2022 and September 2024, which resulted in one additional TT1 patient. To further enrich the dataset, we included data from newborns referred after screening using the NeoBase assay between October 2008 and December 2017 and the FN from 2010. As a result of the enrichment, the dataset contains a substantial number of TT1 patients, which makes it possible to obtain a more reliable PPV, but the enriched dataset does not reflect the Dutch newborn population. However, this introduces no limitation on the analysis, since our aim was to improve the PPV without negatively affecting the sensitivity.

No effect on the performance of the screening protocol was observed after addition of the biomarker phe, the SA/tyr ratio or the tyr/SA ratio. Regarding the ratios, it is plausible that the observed increase in both SA and tyr in TT1 patients is less apparent in a ratio of the two biomarkers, resulting in a less clear discrimination of TPs from TNs (Figure 3). The PPV of the used dataset increases from 9% with the current screening protocol (SA ≥ 0.60 µmol/L) to 72% with an alternative screening protocol combining multiple biomarkers. The inclusion of three or four additional biomarkers is expected to increase the PPV by excluding the subset of samples with only increased SA concentrations, for example due to an analytical issue. This will eliminate the need to repeat the screening following abnormal results and prevent delay in the referral of true patients.

While most international screening protocols appear to be limited to the use of SA and tyr as biomarkers for TT1 [16], the ‘Plot by Condition’ tool with both the ‘unadjusted values’ and the ‘adjustment values’ option in CLIR identifies numerous (ratios of) biomarkers that would discriminate with greater accuracy than the current SA or tyr screening protocols [19]. The ratios included multiple ratios between SA and an acylcarnitine, such as SA/C3 and C10:1/SA. We did not include these ratios in the current analyses, as the focus was on the metabolites involved in the tyr catabolic pathway since we expected these biomarkers to have the highest impact. However, considering the discriminating power reported by CLIR, these ratios may prove beneficial in enhancing the performance of the current approach and could be considered in future studies aimed at further improvements. In 2017, the presumed benign condition maleylacetoacetate isomerase deficiency has been identified as a possible cause of elevated SA in the NBS for TT1 [22]. The quantification of maleic acid in DBSs as a second-tier biomarker could prevent the finding of FP results [3], but this assay is yet to be developed.

Another interesting approach is to use machine learning to identify the optimal combination of biomarkers for the detection of TT1, thereby improving the specificity and PPV (and perhaps the sensitivity and NPV), in an approach similar to that used in the ongoing study in the Netherlands in order to improve the PPV of the congenital hypothyroidism screening protocol [23,24]. Other factors that may play a role in the outcome include the timing of the heel-prick. This was not included in the present study but could be investigated in future studies using a machine learning model. In addition, DNA techniques such as next generation sequencing (NGS) are increasingly incorporated in NBS protocols. Since TT1 is a monogenetic disorder, potential future improvement could be achieved by using NGS or other DNA techniques as one of the tiers in the NBS protocol [16,25]. In a protocol using (a combination of) biomarkers as a first-tier screening with low COVs to guarantee 100% sensitivity, NGS could be employed as second-tier screening to select only those children with a disease-causing variant on the gene encoding for FAH for referral for 100% specificity. Nevertheless, NGS remains a costly option, would possibly delay patient referral and is not currently employed in the Dutch screening programme.

Our current study focused on improving the PPV, but several publications mention TT1 patients with normal or only slightly elevated SA and/or tyr values [5,6,7], resulting in FN cases. This emphasizes that simply lowering the COV for SA or tyr will not prevent FN cases. Rehsi et al. also suggest an alternative biomarker [7], but no DBS screening method has been developed yet, and the sensitivity and specificity of this biomarker are unknown.

Given that the known FN in this study had a converted SA above the current COV, it is not inconceivable that other children with a previous negative screening result, but with a converted SA above the current COV, are in fact undetected FN cases. In a follow-up study, the potential presence of additional FNs will be investigated using a machine learning approach.

In conclusion, in our study, we showed that adding additional biomarkers to our TT1 newborn-screening programme resulted in a higher PPV without loss of sensitivity. This prevents harm to families that are facing an FP referral. It also eliminates the need for re-analysis of the DBSs following an initial increased SA concentration, thus avoiding delays in patient referral due to re-analysis and sometimes resampling. An alternative screening protocol combining SA (≥0.60 µmol/L), tyr (≥100 µmol/L), tyr × SA (≥110 µmol^2^/L^2^) and tyr/phe (≥2.5) resulted in the highest PPV (72%) and classified all thirteen TT1 patients diagnosed between 1 October 2008 and 30 September 2024 (Table 2), including the previously missed patient, as screen-positive. Therefore, we recommend considering the implementation of this protocol in the Dutch NBS programme.

## Figures and Tables

**Table 1:** Comparison of the different screening protocols used in the Dutch programme for TT1, including the performance indicators and prevalences.

Period	1 January 2018/ 31 December 2021	1 July 2009/ 31 December 2017	1 October 2008/ 30 June 2009	1 January 2007/ 28 February 2007
Analyzer	Waters Xevo TQD	Waters Quattro Micro/Premier	Waters Quattro Micro/Premier	Waters Quattro Micro
Assay	Revvity NeoBase 2 non-derivatized kit	Revvity NeoBase non-derivatized kit	Revvity NeoBase non-derivatized kit	Revvity NeoGram non-derivatized kit
COV	SA ≥ 0.60 µmol/L blood *	SA ≥ 1.2 µmol/L blood	SA ≥ 1.5 µmol/L blood	Tyr ≥ 500 µmol/L blood
Screened newborns	693,821	1,506,935	136,644	34,111
TN	693,798	1,506,912	136,641	34,099
FN	0	1	0	0
TP	2	8	1	0
FP	21	14	2	12
Referral rate	0.0033%	0.0015%	0.0022%	0.0352%
Sensitivity	100%	89%	100%	-
Specificity	99.997%	99.999%	99.999%	99.965%
PPV	9%	36%	33%	0%
Prevalence	1:346,910	1:167,437	1:68,322 ^$^	-

* Between 1 January 2018 and 31 March 2019, the official COV was temporarily 0.90 µmol/L blood. ^$^ One known patient was not screened. This resulted in two patients in this period, with one true-positive result and no known false-negative results.

**Table 2:** Overview of biomarker concentrations determined from DBSs of TT1 patients screened between 1 October 2008 and 30 September 2024, including the false-negative case (in italics). For samples analysed with the NeoBase assay, the converted SA concentrations are also shown, and these converted SA concentrations are also used to calculate ratios and the product of tyr and SA. Of the thirteen patients, only five had tyr concentrations above the cut-off value used in 2007 (≥500 µmol/L, in bold). The righthand column contains the original screening result based on the SA concentration and the SA cut-off value used at the time of screening, which are displayed in the fourth and third columns, respectively.

	Screening Assay	SA Cut-Off Value µmol/L	SA µmol/L	Converted SA ^#^ µmol/L	tyr µmol/L	phe µmol/L	tyr × SA ^#^ µmol^2^/L^2^	tyr/SA ^#^	SA ^#^/tyr	SA ^#^/phe	tyr/phe	phe/tyr	Original Screening Result
1	NeoBase	1.50	4.93	2.84	**829**	81	2354	292	0.003	0.035	10.23	0.10	Positive
2	NeoBase	1.20	4.37	2.52	439	78	1106	174	0.006	0.032	5.63	0.18	Positive
*3*	*NeoBase*	*1.20*	*1.08*	*0.64*	*288*	*68*	*184*	*450*	*0.002*	*0.009*	*4.24*	*0.24*	*Negative*
4	NeoBase	1.20	6.70	3.85	**833**	137	3207	216	0.005	0.028	6.08	0.16	Positive
5	NeoBase	1.20	7.96	4.57	416	55	1901	91	0.011	0.083	7.56	0.13	Positive
6	NeoBase	1.20	5.13	2.95	493	47	1454	167	0.006	0.063	10.49	0.10	Positive
7	NeoBase	1.20	5.48	3.15	**500**	60	1575	159	0.006	0.053	8.33	0.12	Positive
8	NeoBase	1.20	1.90	1.11	**900**	64	999	811	0.001	0.017	14.06	0.07	Positive
9	NeoBase	1.20	16.93	9.70	236	56	2289	24	0.041	0.173	4.21	0.24	Positive
10	NeoBase	1.20	5.32	3.06	**569**	75	1741	186	0.005	0.041	7.59	0.13	Positive
11	NeoBase 2	0.60	0.99	N/A	112	43	111	113	0.009	0.023	2.60	0.38	Positive
12	NeoBase 2	0.60	2.92	N/A	422	30	1232	145	0.007	0.097	14.07	0.07	Positive
13	NeoBase 2	0.60	2.03	N/A	465	87	944	229	0.004	0.023	5.34	0.19	Positive

^#^ SA concentration obtained with the NeoBase assay converted to the level of the NeoBase 2 assay using the equation NeoBase 2 = 0.019 + 0.572 × NeoBase. If the initial SA concentration was determined using the NeoBase assay, the converted SA concentration was used to calculate ratios and products.

**Table 3:** Performance of current and alternative screening protocols. Overview of current and alternative screening protocols and the calculated effect of these screening protocols, maintaining 100% sensitivity, on the PPV. The analysis is based on data from January 2018 up to and including December 2022, and the dataset was enriched with data from the false-negative case and from referred newborns from October 2008 up to and including December 2017, as well as that from newborns referred for TT1 screening between 1 January 2022 and 30 September 2024. Only the three alternative screening protocols with the highest PPVs are shown. A complete list of alternative screening protocols with PPVs higher than that of the current screening protocol are shown in the Supplementary Materials. Because of the enrichment of the datasets with TPs, FPs, and FN from periods for which the TN datasets have not been included, the enriched dataset does not reflect the true Dutch newborn population, and the calculated specificity and NPV cannot be transferred to the Dutch newborn population (TN = true negative, FN = false negative, TP = true positive, FP = false positive, PPV = positive predictive value, NPV = negative predictive value).

Protocols and Cut-Off Values	TN	FN	TP	FP	Referral Rate (%)	Sensitivity (%)	Specificity (%)	PPV (%)	NPV (%)
Current protocol SA ≥ 0.60 µmol/L	653,396	0	13	127	0.0214	100	99.981	9	100
Alternative L SA ≥ 0.60 µmol/L Tyr ≥ 100 µmol/L Tyr × SA ≥ 100 µmol^2^/L^2^ Tyr/Phe ≥ 2.5	653,515	0	13	8	0.0032	100	99.999	62	100
Alternative N SA ≥ 0.60 µmol/L Tyr ≥ 100 µmol/L Tyr × SA ≥ 110 µmol^2^/L^2^ Phe/Tyr ≤ 0.5	653,516	0	13	7	0.0031	100	99.999	65	100
Alternative O SA ≥ 0.60 µmol/L Tyr ≥ 100 µmol/L Tyr × SA ≥ 110 µmol^2^/L^2^ Tyr/Phe ≥ 2.5	653,518	0	13	5	0.0028	100	99.999	72	100

## Data Availability

Data presented in this study is not publicly available due to privacy restrictions and access can only be requested at the appropriate committees within the Dutch programme as described in Section 2.2. Procedures are described on https://www.pns.nl/hielprik/professionals/opvragen-data-en-bloed (in Dutch).

## Supplementary Materials

The following supporting information can be downloaded at: https://www.mdpi.com/article/10.3390/ijns11020035/s1, Table S1: Performance of current and alternative screening protocols.

## Abbreviations

The following abbreviations are used in this manuscript:

CDC	Centers for Disease Control and Prevention
COV	Cut-off value
DBS	Dried blood spot
FIA-MS/MS	Flow-injection analysis tandem mass spectrometry
FN	False negative
FP	False positive
HCC	Hepatocellular carcinoma
NBS	Newborn screening
NGS	Next-generation sequencing
NPV	Negative predictive value
Phe	Phenylalanine
PKU	Phenylketonuria
PPV	Positive predictive value
QC	Quality control
SA	Succinylacetone
TP	True positive
TT1	Tyrosinemia type 1
tyr	Tyrosine
