# Supplementary material for: Evaluation of the Performance of Newborn Screening for Tyrosinemia Type 1 in The Netherlands: Suggestions for Improvements Using Additional Biomarkers in Addition to Succinylacetone
Source: Int J Neonatal Screen. 2025 May 9;11(2):35. doi: 10.3390/ijns11020035 (PMC12101382; doi:10.3390/ijns11020035)
Supplement: Supplementary file 1 [file IJNS-11-00035-s001.zip › IJNS-3606806-supplementary.pdf]

Evaluation of the Performance of Newborn Screening for Tyrosinemia Type 1 in the Netherlands: Suggestions for Improvements Using Additional Biomarkers in Addition to Succinylacetone

Marelle J. Bouva, Allysa M. Kuypers, Evelien A. Kemper, Rose E. Maase, Annet M. Bosch, Francjan J. van Spronsen, Annemieke C. Heijboer, M. Rebecca Heiner-Fokkema, Sandra G. Heil and Anita Boelen

Supplementary Material.

Table S1. Performance of current and alternative screening protocols.

Overview of current and alternative screening protocols and the calculated effect of these screening protocols, maintaining a 100% sensitivity, on the PPV using data from January 2018 up to and including December 2022, enriched with data of the referred newborns and the false negative newborn from October 2008 up to and including December 2017 and the data from newborns referred for TT1 screened between 1 January 2022 and 30 September 2024. Only alternative screening protocols with a higher PPV than the current screening protocol are shown. Because of the enrichment of the datasets with TP, FP, and FN from periods for which the TN datasets have not been included, the composed dataset does not reflect the Dutch newborn population, and the calculated specificity and NPV can not be transferred to the Dutch newborn population. (TN = true negative, FN = false negative, TP = true positive, FP = false positive, PPV = positive predictive value, NPV = negative predictive value)

Protocols and cut-off values	TN	FN	TP	FP	Referral rate (%)	Sensitivity (%)	Specificity (%)	PPV (%)	NPV (%)
**Current protocol**<br>**SA ≥0.60 µmol/L**	653,396	0	13	127	0.0214	100	99.981	9	100
**Alternative A**<br>**Tyr × SA ≥110 µmol^2/L^2**	653,426	0	13	97	0.0168	100	99.985	12	100
**Alternative B**<br>**SA ≥0.60 µmol/L**<br>**Tyr ≥100 µmol/L**	653,481	0	13	43	0.0086	100	99.993	23	100
**Alternative C**<br>**SA ≥0.60 µmol/L**<br>**Tyr ≥100 µmol/L**<br>**Phe ≥25 µmol/L**	653,481	0	13	43	0.0086	100	99.993	23	100
**Alternative D**<br>**SA ≥0.60 µmol/L**<br>**Tyr ≥100 µmol/L**<br>**Tyr × SA ≥90 µmol^2/L^2**	653,504	0	13	19	0.0049	100	99.997	41	100
**Alternative E**<br>**SA ≥0.60 µmol/L**<br>**Tyr ≥100 µmol/L**<br>**Tyr × SA ≥95 µmol^2/L^2**	653,506	0	13	17	0.0046	100	99.997	43	100

Protocols and cut-off values	TN	FN	TP	FP	Referral rate (%)	Sensitivity (%)	Specificity (%)	PPV (%)	NPV (%)
**Alternative F**<br>**SA ≥0.60 µmol/L**<br>**Tyr ≥100 µmol/L**<br>**Tyr × SA ≥100 µmol^2/L^2**	653,510	0	13	13	0.0040	100	99.998	50	100
**Alternative G**<br>**SA ≥0.60 µmol/L**<br>**Tyr ≥100 µmol/L**<br>**Tyr × SA ≥100 µmol^2/L^2**<br>**Phe ≥25 µmol/L**	653,510	0	13	13	0.0040	100	99.998	50	100
**Alternative H**<br>**SA ≥0.60 µmol/L**<br>**Tyr ≥100 µmol/L**<br>**Tyr × SA ≥100 µmol^2/L^2**<br>**SA/Phe ≥0.0090**	653,512	0	13	11	0.0037	100	99.998	54	100
**Alternative I**<br>**SA ≥0.60 µmol/L**<br>**Tyr ≥100 µmol/L**<br>**Tyr × SA ≥100 µmol^2/L^2**<br>**Phe/Tyr≤0.5**	653,513	0	13	10	0.0035	100	99.998	57	100
**Alternative J**<br>**SA ≥0.60 µmol/L**<br>**Tyr ≥100 µmol/L**<br>**Tyr × SA ≥110 µmol^2/L^2**	653,514	0	13	9	0.0034	100	99.999	59	100
**Alternative K**<br>**SA ≥0.60 µmol/L**<br>**Tyr ≥100 µmol/L**<br>**Tyr × SA ≥110 µmol^2/L^2**<br>**Phe ≥25 µmol/L**	653,514	0	13	9	0.0034	100	99.999	59	100
**Alternative L**<br>**SA ≥0.60 µmol/L**<br>**Tyr ≥100 µmol/L**<br>**Tyr × SA ≥100 µmol^2/L^2**<br>**Tyr/Phe ≥2.5**	653,515	0	13	8	0.0032	100	99.999	62	100
**Alternative M**<br>**SA ≥0.60 µmol/L**<br>**Tyr ≥100 µmol/L**<br>**Tyr × SA ≥100 µmol^2/L^2**<br>**Tyr/Phe ≥2.5**<br>**SA/Phe ≥0.0090**	653,515	0	13	8	0.0032	100	99.999	62	100
**Alternative N**<br>**SA ≥0.60 µmol/L**<br>**Tyr ≥100 µmol/L**<br>**Tyr × SA ≥110 µmol^2/L^2**<br>**Phe/Tyr≤0.5**	653,516	0	13	7	0.0031	100	99.999	65	100
**Alternative O**<br>**SA ≥0.60 µmol/L**<br>**Tyr ≥100 µmol/L**<br>**Tyr × SA ≥110 µmol^2/L^2**<br>**Tyr/Phe ≥2.5**	653,518	0	13	5	0.0028	100	99.999	72	100
